# Monoclonal Antibodies Addressed to Factors of Signalization in Keloid Scars: Opportunities and Areas of Action

**DOI:** 10.7759/cureus.8894

**Published:** 2020-06-28

**Authors:** Erick Moreno Pizarro, Eduardo Morales Valencia, Arturo Pérez Cuéllar, Camilo Acuña Pinzon, Aarón Emanuel Serrano Padilla

**Affiliations:** 1 Medicine, University of Guanajuato, León, MEX; 2 Surgery, Hospital Regional de Alta Especialidad del Bajío, León, MEX; 3 Orthopedics and Traumatology, General Hospital of León, León, MEX; 4 Orthopedics and Traumatology, Medica Campestre Hospital, León, MEX; 5 General Surgery, Hospital Regional de Alta Especialidad del Bajío, León, MEX

**Keywords:** keloid scar, monoclonal antibodies, abnormal scarring, aesthetic, genetics, scar appearance

## Abstract

The advance of technology has made possible the use of new techniques within medicine for the treatment of diseases; monoclonal antibodies are a clear example of this. Keloid scars are one of the most difficult pathologies to treat due to the high percentage of recidivism, formed by the growth of a scar with benign fibrous tissue in genetically predisposed individuals, resulting from a process of inflammation and abnormal scarring. Monoclonal antibodies, being a line of treatment that has increased over the years, can show a new frontier in the treatment of them by focusing on the signaling that causes it. We review the literature on the signaling mechanisms of keloid scars and the possible monoclonal approach.

## Introduction and background

The origin of monoclonal antibodies lies through the research work of Georges Köhler and César Milstein in 1975 [1]. They sought to reproduce a cell line with the ability to maintain stability by secreting immunoglobulins against specific antigens indefinitely, obtained from the union of two cells through centrifugation with polyethylene glycol, a method that combines a chemical and a physical medium. Its first historical use dates back to 1982 when it was used as therapy for lymphoma. At the same time, recognition of the hyperactive effect and tolerance to human anti-murine antibodies led to the development of the process of chimerization and humanization. Monoclonal antibodies have a structure of specialized glycoproteins capable of recognizing epitopes and developing the marking or target required for the desired purpose, among molecular biology, biotechnology, treatment of diseases, and many others [2-3].

The potential benefits through the action of these defense mechanisms can be exploited with the creation of antibodies capable of recognizing epitopes of interest for study and research. Keloid healing is a benign tumor of fibrous nature characterized by abnormal and unregulated growth of dermal fibroblasts, irregular deposition of glycosaminoglycans around the wound, low levels of hyaluronic acid, and overproduction or alteration of the extracellular matrix [4-5].

Its main difference with hypertrophic scars is the potential invasion and infiltration of surrounding tissues and an excessive expansion without quiescent periods and without a regressive phase or remodeling (they continue to develop at different speeds). Currently, the genetic factors behind the keloid scar remain under investigation. Darker-skinned individuals have a greater predisposition than lighter-skinned individuals [6]. Individuals of African origin or descent have the highest prevalence rate of 4% - 6%; however, in the adult population of Zaire, it is 16%. Asian and Hispanic descendants are less predisposed, and Caucasians have the lowest prevalence rate (as low as 0.09% in England).

## Review

Antibody structure

Antibodies are the surface structure of a biochemical mechanism of interaction capable of recognizing and developing actions against certain antigen epitopes. This system is made up of two heavy chains (CH) and two light chains (CL), joined by the force of disulfide bridges that will compose the antigenic recognition (Fab) and crystallizable fraction (Fc) systems in charge of antibody-dependent cell cytotoxicity (ADCC) and complement-mediated cytotoxicity (CD) [3]​​​​​​.

Development of monoclonal antibodies

The technology created by Georges Köhler and César Milstein involved immunizing a B cell of an animal previously inoculated with the antigen of importance to be developed to produce the clones with the specific determinants for the antigen. The second step of interest was to extract a cell from tumor tissue, specifically, from myeloma that was unable to secrete antibodies deficient of the hypoxanthine-guanine-phosphoribosyltransferase (HGPRT) enzyme. The immune power of the B cell was obtained by binding these two cells together to the virtually infinite division capacity of monoclonal antibodies (directed to a single epitope) or polyclonal antibodies (directed to different epitopes) whose resulting cell was named "hybridoma." Once the hybridomas were obtained, they were placed in a special composition culture medium of hypoxanthine-aminopterin-thymidine (HAT) which allowed only the development of the hybridomas and was ineffective for the survival of the B cell and the myeloma. It was tested for effectiveness, selecting only the desired ones, and verified their specificity by binding to the previously known antigen. Then, by the limit dilution method, cloning was performed, storing and conserving them in dimethyl sulfoxide indefinitely. The hybridomas developed by Köhler and Milstein had complications since they preserved murine regions, which developed a response in the human immune system and generated tolerance to their effect. Therefore, a process called chimerization was developed in 1984: preserve only the variable murine regions and the rest of the heavy and light chains remain of human origin. Later, the process of humanization where only the hypervariable regions of the light and heavy chains were of murine origin and the rest of human origin were developed. The use of monoclonal antibodies linked to compounds, enzymes, and pro-drugs opened a wide field of innovations and possibilities on targeted treatments [2-4].

Keloids

The term keloid was originally portrayed in the 19th century as "cheloid," acquired from the Greek word "chile," translated to "crab claw" [7].

According to the Gauglitz et al. study published in 2011, 100 million patients developed exacerbated healing each year of which 55 million required some treatment quickly to avoid future complications [8]. Like the Mamalis et al. study, it was calculated that approximately 4.5% to 16% of the population of Hispanic or African descent suffered from keloids or hypertrophic scars [9]. The keloid lesion was also classified in different aspects, such as Tirgan's area [10].

Keloid development

The wound healing process is made up of four fundamental processes (hemostasis, inflammation, proliferation, and remodeling) which are mediated through the presence of growth factors and cytokines (Table 1) [11-15].

**Table 1:** Scarring Mechanism IL-1: interleukin 1

Hemostasis	Inflammation	Proliferation	Remodeling and Maturation
The period prior to inflammation, mentioned by some authors and present in all subjects, as it is a normal process of healing [15].	48 - 72 hours after injury, being initiated by the release of IL-1 by keratinocytes	Lasts from 6 to 8 weeks (2 months approximately)	Mediated through the metalloproteinases, it is the limiting and fundamental step that has been occluded in the keloids; in charge of granting the structure and coupling to the surrounding tissue, its main medium is the presence of Smad 7.

Apart from wound trauma, the main risk factors for keloid development include age between 10 to 30 years, blood type A, hormonal spikes in puberty and pregnancy, Hispanic, African American, or Asian race, as well as hyperimmunoglobulinemia E [11-12]. The main sites of appearance are trunk, ears, back, and chest [14].​​​​​​

Keloid scars are mainly composed of type 1 and 3 collagen (COL1 and COL3 families) grouped in variable thicknesses with disorganized limits and with an excess of myofibroblasts, whose pathological history culminates in the overexpression of fibroblastic proteins and subsequent failure in the change to the last phase of tissue repair (remodeling) [16].

Various researchers have reported the presence of mast cells and histamine in keloid tissue, which explains the origin of the pruritus in an early stage [13]. However, the presence of the development of keloids ranges from one to seven years after the injury has been denoted, despite the fact that it is common to appear three months after the injury [13-14].

Mancini et al. and Peacock et al. developed a classification between keloid and hypertrophic scars in the years 1962 and 1970, respectively [17-18]. Within their classification, both have growth above the level of the skin. However, as previously mentioned, their main difference was the ability of infiltration and invasion of surrounding tissues in the keloids [19].

Signaling factors associated with keloid scarring

Most of the transforming growth factor-β (TGF-β) family have been described, as well as SMAD (second messenger) proteins, P53, epidermal growth factor receptor (EGFR), mTOR signaling pathways, along with the WNT5a / β catenin signaling pathway [20]. The key role of the TGF-β family should be highlighted. Studies have shown that it triggers the disease by being poorly regulated. Therefore, antibodies against them have been developed, such as anti-TGF-β1 and anti-TGF-β2, which suppress the synthesis of collagen from keloid fibroblast cultures and human keloid derived xenograft collagen, demonstrating its role in the mechanism of the disease [4].

In the same way, TIEG-1 (TGF-β inducible early gene) is involved in this process way before the expression of the TFG-β family by binding and repressing Smad7 (responsible for the negative feedback of TGF- β / Smad) in keloids, and therefore, the subsequent overexpression of TIEG-1 and its TGF-β family release chain [21].​​​​​​

New barriers in signaling

mTOR

The mammalian target of rapamycin (mTOR) is a protein involved in the development of micro ribonucleic acid (miRNA) transcription and regulation of growth, proliferation, and cell death. It can generate two signaling complexes (which we will not discuss further). mTORC1 (binds to the regulatory associated protein of mTOR (RAPTOR)) and mTORC2 (binds to the rapamycin-insensitive companion of mammalian target of rapamycin (RICTOR)). In keloid scars, the levels of endogenous mTOR, phosphorylated mTOR, and p70S6K are elevated, which exerts a symbolic effect on the activation pathway of platelet-derived growth factor (PDGF) and fibroblast growth factor 9 (FGF-9). Together, mTOR and TGF- β1 induce the ADAM12 gene.

miRNAs and lncRNAs

Functional non-coding RNAs, such as microRNAs (miRNAs) and long non-coding RNAs (lncRNAs), participate as key mechanisms in the expression of genes involved in the regulation of the disease. Among them, 54 miRNAs have been identified, whose length varies from 20 to 24 nucleotides [22].

New barriers are found in the following miRNAs:

miR-21: It is considered an oncogenic gene and has been found in high quantities in the development of fibrosis. It plays a vital role in the proliferation of fibroblasts and the overproduction of the extracellular matrix through the phosphatidylinositol-3-kinase/protein kinase B (PI3K/AKT) pathway by regulating PTEN (phosphatase and tensin homolog on chromosome 10) and PDCD4 (programmed cell death 4). It can also affect the expression of the Fas ligand (FasL) in the presence of TGF-β1. Another study showed its inhibitory effect on Smad7 which regulates collagen synthesis in the TGF-β1 pathway [23].

miR-29: miR-29 plays an important role in several fibrotic diseases, such as myocardial fibrosis and pulmonary fibrosis. It can be detected in serum as a biomarker in these diseases. Its relationship with keloids has been demonstrated, but more scientific studies are still required. It is used as a prognosis for the development of the disease. It directly activates TGF-β1, the WNT pathway, and the frizzled protein, culminating in the synthesis of collagen in keloids, mainly COL1 and COL3 [24].

miR-200c: It has been demonstrated that it is capable of reversing the transition process that induces TGF-β1 in different fibrocyte tumors in the epithelium-mesenchyme transition [25]​​​​​​. Research showed that its expression in keloids is 6.92 times lower than in healthy tissue. In regular values, it acts by inhibiting collagen synthesis and cell proliferation previously activated by TGF-β1. Also, the phosphorylation of Smad 2 and Smad 3 signaling was markedly reduced by treatment of exogenous miR-200c; this strongly demonstrates that the loss of miR-200c expression has a vital role in the development of the disease [22].

Biomolecular marker of the disease

A particularly important cell-surface proteoglycan that has recently been used as a potential biomarker is syndecan-1 (CD 138), which is secreted by cells into the extracellular matrix; it is present in neonate scars and absent in fetal tissue repair. It binds to the collagen and fibronectin deposited during the tissue repair process, which allows later reversal of the process and the tissue to enter a quiescent state (absent in keloids), allowing for proper tissue repair [26].

Current treatments for keloid scarring

Currently, there is a wide range of aesthetic treatments against keloid scarring, most of which focus on the destruction of tissue, along with the use of steroid therapies (Table 2). 

**Table 2:** Treatment Options for Keloid Scar

Treatment Option	Description
Triamcinolone (corticosteroid)	This is the first-line treatment to treat keloids. It is used as an intralesional injection and favors a drastic reduction in size (in some cases, excessive, if not administered properly). The dosage can range from 10 - 40 mg. Some of the side effects can be skin atrophy and telangiectasias. The more timely and promptly steroids are administered in higher concentrations, the better and faster the response will be. However, side effects, such as hyperpigmentation and tissue atrophy, tend to occur [27].
Cryotherapy	Liquid nitrogen is commonly used to treat keloids, Meymandi et al. carried out a clinical trial where cryotherapy sessions of 10 seconds with liquid nitrogen was performed using 1 mg/cm^2^ triamcinolone acetonide injection mixed with lidocaine (50:50 ratio), showing a treatment response rate of 75% [28]. The most common adverse effect reported was hyperpigmentation.
Stress-free excision	Z-plasty and W-plasty may be done; however, the recurrence rate is 45% to 100% without combined therapy. The proper excision and repair of the wound are hindered by multiple factors still under study, such as the technique performed, the surgeon's experience, and the starting point of the excision, among others [29].
Radiation	Superficial x-rays, electron-beam therapy, and low- or high-dose-rate brachytherapy have good results in scar reduction. The effects of radiation are thought to mediate inhibition of neovascular buds and proliferating fibroblasts, which result in decreased collagen production. Electron beam irradiation is usually started shortly after keloid excision, and the total dose is limited to 40 Gy throughout several administrations to prevent side effects. Carcinogenesis induced by radiation is widely known, particularly in areas such as the breast and thyroid; its use should be handled with caution [8]. There are reports that have achieved a 95.5% non-recurrence rate based on a combined treatment of excision and administration of platelet-rich plasma with subsequent surface radiation with photons [14]. Within the same study, the greatest effectiveness of the radiotherapy treatment is 72 hours after its appearance or excision.
Laser and light-based therapy	Ablative laser treatment results in keloid tissue destructions, non-ablative lasers, and light-based therapies result in a reduction in size, erythema, and pliability. Different outcomes and recurrence are likely due to variations in lasers, settings, and treatment protocols [9].

**Figure 1:**
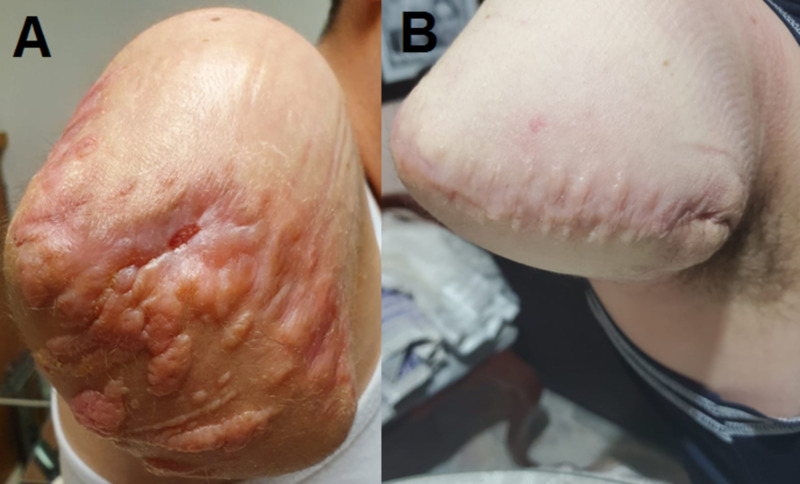
Aesthetic evolution of three months after treatment against keloid scars A: Abnormal scar, three months after surgical excision of the right upper limb; B: aesthetic result once the keloid tissue was surgically removed, followed by three months of treatment using topical steroid infiltration and moisturizing dressings

​​​​​​​​​Despite this, the current treatment approach only works by giving an aesthetic result to the patient but not by blocking the signaling pathways that biochemically generate abnormal scars. Medica Campestre Hospital in León, Mexico, where treatment after surgical excision consists of topical steroid infiltration and moisturizing dressings, shows a favorable evolution for patients. However, the mechanism by which they first appear is not resolved. Hypertrophic scarring is still observable, although with better cosmetic and functional results (Figure 1).

New strategies and clinical use

As the first oral multi-kinase inhibitor, sorafenib blocks multiple intracellular signaling pathways involving keloid signaling. In 2016, Wang et al. conducted a controlled study to observe the interaction of sorafenib, in which it was shown to act as an effective inhibitor of transforming growth factor-beta (TGF-β)/Smad and mitogen-activated protein kinase/extracellular-signal-regulated kinase (MAPK/ERK) in vitro [28]. This resulted in decreased expression of connective tissue growth factor (CTGF), interleukin-6 (IL-6), and interleukin-8 (IL-8), preventing keloid fibroblast migration and proliferation, as well as angiogenesis and collagen accumulation, which presented as the first step of favorable results by inhibiting keloid healing signaling pathways [30]​​​​​​.

## Conclusions

Given the enormous possibility of pathways involved in the signaling of keloid pathology, a wide range of experimentation is opening up, although analogs and blockers (among others) can be used.

We highlight the emphasis on signaling through TGF-β and the key role of Smad7 within this range of simultaneous and complex processes. The signaling of the TGF-β family has not been fully unraveled within the pathology, and the use of antibodies will greatly favor the detection of unknown factors and a better understanding of their behavior. It is worth mentioning the decisive role played by miRNAs (having as exponents miR-21 and miR-200c) favor a wider and more committed view of the environmental and biochemical factors that predispose to such conditions. Based on the present work for future applications in the development and understanding of multifactorial vectors of disease development, it will be used as a basis for the possible development of such monoclonal antibodies directed to keloid growth factors, defined as the main vector of importance, the expression of the TGF- β family, and its surroundings within the signaling cascade.
